# Annotations

**Published:** 1908-03-07

**Authors:** 


### Annotations.

### Viewing the Body.

At the Southwark Coroner's Court, on February 18, Dr. F. J. Waldo, the City Coroner, referred to the Bill which passed its first reading in the House of Commons last session, to dispense with the compulsory viewing of the body by coroners' juries, except on the order of the coroner or when a majority of the jury require it. Dr. Waldo gives it as his emphatic opinion that this often unpleasant duty should be retained in the public interest. The viewing of the body, in his opinion, acts as a deterrent to crime, and provides some of the best evidence that can be adduced, especially in cases of neglect, whilst it assists juries to follow the evidence more intelligently. He maintains that the view of the body is the fundamental principle of an inquest. Dr. Waldo further states that the majority of those coroners who, like himself. give their whole time to the work and hold the largest number of inquests, are in favour of retaining the present system. This defence of an ancient practice should be weighed carefully by Members of Parliament before the second reading of the Bill. It would be a pity to abolish the compulsory viewing of the body merely for the sake of a small economy in time and money unless it can be shown that the custom is unnecessary and obsolete.

### What are Spirits?

The first public sitting of the Royal Commission on Whisky and other Potable Spirits was entirely given up to the examination of Mr. A. J. Tedder, Chief Inspector of Excise. The witness, at the request of the Commissioners, went into the details of spirit manufacture in the United Kingdom. With regard to the materials from which spirits are made, British distillers use malt, unmalted grain—including maize, barley, oats, rye, and a little wheat—molasses, rice, glucose, and various other substances, such as sugar, sago, tapioca, bran, and malt combings. There is no legal restriction as to the materials from which spirits may be manufactured; and, except in the case of molasses, the Excise officers exercise no control over the ingredients. In Scotland, out of 150 distilleries now working, 142 use malt only, and 137 use only pot-stills. In patent still distilleries maize is principally used. The total number of active distilleries in the three kingdoms is 185, and their total output last year was 50,317,908 gallons, representing a total duty of £17,745,125. With regard to official supervision, it is not until fermentation has begun that the Excise officers begin to control the distiller's procedure. All subsequent operations, including blendings and other warehouse operations, are directly supervised. Distillers may bond spirit at any strength between 20 under-proof and 25 over-proof. They may "reduce" the spirit with water in preparing it for customers, and they may add fluid colouring matter to the extent of one pint in eighty gallons. During distillation no flavouring ingredient may be added. With regard to the two processes of manufacture, patent-still spirit is the cheaper to produce. Moreover, it is kept a shorter time in bond than pot-still spirit, while from patent stills the spirit runs continuously at a much higher strength than from pot-stills. Mr. Tedder stated that there was no essential distinction between Irish and Scotch pot-still whiskies. In reply to a Commissioner, he said that there was no official definition of whisky; it was not differentiated as such from other spirits in the returns.

### Hospital Appointments and the M.R.C.S. Diploma

It will be remembered that at the annual meeting of the Fellows and members of the Royal College of Surgeons of England a resolution was carried asking the Council of the College to use its moral influence with hospital authorities to recognise members of the College as having equal rights with provincial, Scottish, and Irish graduates, in their application for hospital appointments. At the last meeting of the Council this resolution was considered, and it was decided to circulate a memorandum on the subject among the hospitals of England and Wales. This report draws attention to the returns of the examinations for the Royal Naval Medical Service, the Royal Army Medical Corps, and the Indian Medical Service during the last five years, and shows that candidates holding the diplomas of M.R.C.S.Eng. and L.R.C.P.Lond. have obtained a very high percentage of passes, and that in twenty-seven examinations they gained the first place on eighteen occasions. It will be interesting to see what will be the effect of this official memorandum upon the selection of candidates by hospital committees in the provinces and elsewhere. There seems little doubt that, on certain occasions in the past, candidates holding the English conjoint diplomas have been at an undeserved disadvantage.

### The late Mr. Reginald Harrison.

We regret to have to record the death, on Friday, February 28, of Mr. Reginald Harrison, F.R.C.S., at the age of 70. Mr. Harrison's name is prominently associated with the surgery of the genito-urinary system: on this subject he wrote several important works, in addition to a large number of papers contributed to current medical literature. His last article in The Hospital was published only five weeks ago. Mr. Harrison was educated at Rossall School and St. Bartholomew's Hospital, and during a busy life he held numerous professional appointments. He was successively Hunterian Professor, member of Council, and Vice-President of the Royal College of Surgeons of England. He was consulting surgeon to St. Peter's Hospital for Stone, and had been surgeon to the Liverpool Royal Infirmary, and Lecturer on Clinical Surgery at Victoria University. Mr. Harrison took the deepest interest in all matters connected with the ambulance service. He was a Knight of Grace of the Order of St. John of Jerusalem, and president of the Metropolitan Street Ambulance Association. His loss will be felt by a wide circle of friends and colleagues.

